# Spontaneous coronary artery dissection and proximal LAD stenosis in a young male with HIV: A case report

**DOI:** 10.34172/jcvtr.025.33262

**Published:** 2025-12-17

**Authors:** Stella-Maria Kyvelou, Konstantinos Kalogeras, Nikolaos Vythoulkas-Biotis, Panteleimon Pantelidis, Georgios Angelos Papamikroulis, Manolis Vavuranakis

**Affiliations:** 3rd Department of Cardiology, School of Medicine, National and Kapodistrian University of Athens, Thoracic Diseases Hospital of Athens “Sotiria”, Athens, Greece

**Keywords:** Human immunodeficiency virus, Acquired immune deficiency syndrome, Antiretroviral therapy, Myocardial infarction, Spontaneous coronary artery dissection

## Abstract

HIV/AIDS patients under antiretroviral therapy have increased cardiovascular risk. Spontaneous coronary artery dissection, an uncommon yet recognized cause of acute coronary syndrome, typically affects middle-aged females, especially during pregnancy. We present a rare case of SCAD and proximal left anterior descending stenosis in a 45-year-old male with HIV.

## Introduction

 Spontaneous Coronary Artery Dissection (SCAD) is a distinct and uncommon cause of non-atherosclerotic myocardial infarction (MI) occurring in the middle-aged population, with a higher incidence observed among women during the peripartum period. SCAD is estimated to account for less than 1% of all acute myocardial infarctions in both sexes.^1^ It occurs when a hematoma forms within the middle layer of the coronary artery wall, creating a false lumen.^1,2^ The precise underlying causes remain unknown, however, it has been linked to emotional and physical stress, pharmaceutical substances, hormonal stimuli, as well as various inflammatory conditions.^1^ Human immunodeficiency virus (HIV) and antiretroviral therapy have immediate effects on coronary arteries through a variety of mechanisms, such as endothelial dysfunction, vascular injury, and progression of atherosclerosis.^3^ We report the case of a 45-year-old male with a diagnosis of HIV, under antiretroviral therapy who presented with an incident of SCAD along with a stenosis of the proximal left anterior descending artery (LAD).

## Case Presentation

 A 45-year-old male was admitted to our hospital with ongoing chest pain. Upon admission, his blood pressure was 120/70mmHg, and his heart rate was 100bpm. His electrocardiogram revealed no significant changes and his peak troponin levels were 513.4 pg/mL. Point-of-care ultrasound (POCUS) was performed and showed a normal dimension left ventricle with normal systolic function (EF = 60%) and right ventricular systolic pressure (RVSP): 25-30mmHg.

 As for cardiovascular risk factors, our patient was an active smoker and positive for HIV infection, under highly active antiretroviral therapy (HAART) with Dolutegravir/Abacavir/Lamivudine. Upon his arrival at our center, he reported that he had an undetectable viral load. He had no other risk factors of note.

**Figure 1:**
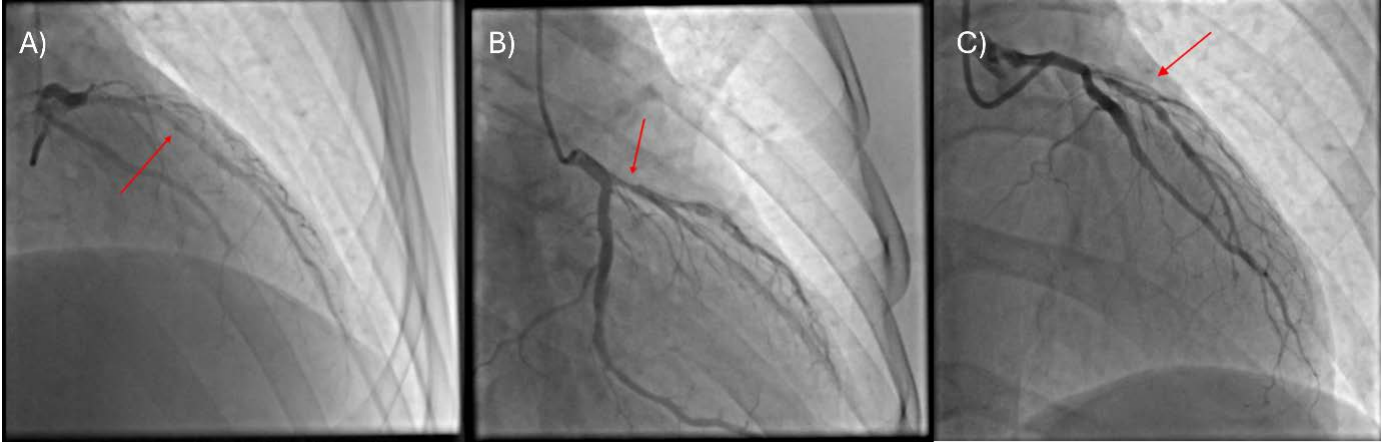
A) Initial Coronary angiography showing the dissected area of the vessel, B) the proximal LAD stenosis, C) Follow-up angiography 5-days later showing signs of healing

**Figure 2:**
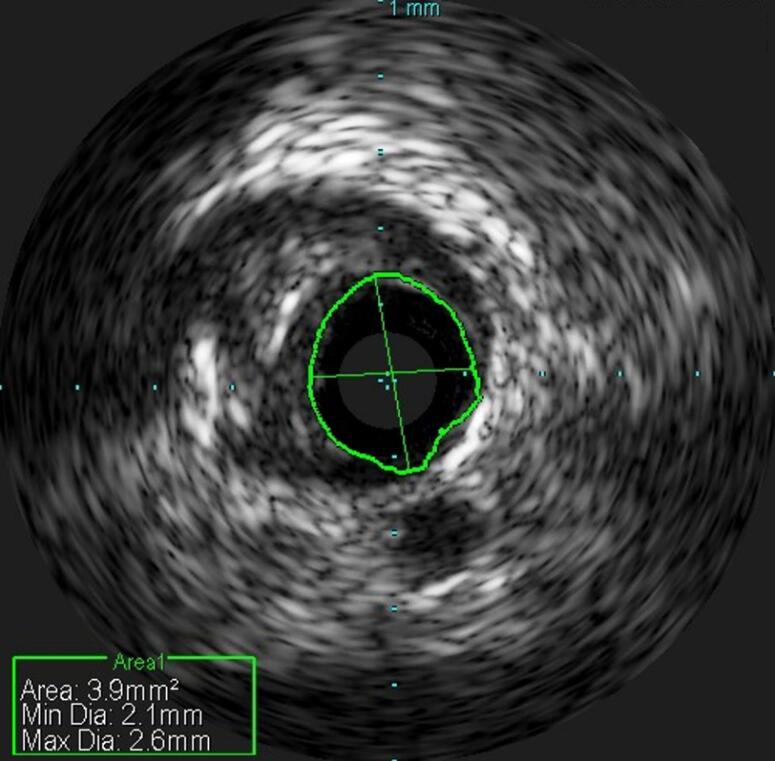
Intravascular ultrasound showing the area of the dissection

 He underwent a coronary angiography which revealed a moderate stenosis in proximal LAD as well as an elongated intracoronary clarification extending from the proximal to the middle segment of the vessel with normal distal flow, TIMI III (Figure 1A-B), (Supplementary Files, Video 1 - 2). We performed an intravascular ultrasound (IVUS) which confirmed the coronary artery dissection with a length of 3.9mm^2^ (Figure 2).

 After the first angiography, no intervention was performed, and he was treated with dual antiplatelet therapy (DAPT) (Aspirin and Clopidogrel). Five days later we performed another coronary angiography, which revealed signs of healing of the dissected vessel area (Figure 1C), (Supplementary Files, Video 3). Simultaneously, an instantaneous wave-free ratio (IFR) was performed, to assess the severity of the proximal LAD lesion, which was not significant in three consecutive measurements (IFR = 0.96, 0.95, 0.96). The patient was discharged with instructions to continue DAPT and reevaluation of the dissection with a future angiography. At the same time, his HIV treatment changed to Doravirine/Tenofovir/Lamivudine, due to increased cardiovascular risk caused by the current antiretroviral therapy.

**Figure 3:**
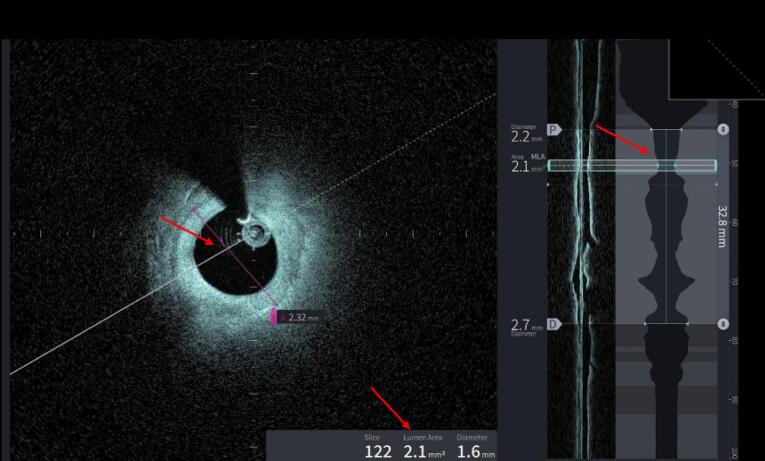
OCT of the dissected area and the proximal stenosis at the follow-up angiography

 5 months later a new coronary angiography and an optical coherence tomography (OCT) were performed to evaluate the healing of the dissection site (Supplementary Files, Video 4). Coronary angiography revealed a decreased intracoronary clearance at the site of the dissection and improved angiographic image in the presence of mild calcification (Supplementary Files, Video 5). OCT revealed severe proximal LAD stenosis (MLA: 2.1mm^2^) (Figure 3).

 The patient (symptom-free) was once again discharged with instructions for DAPT continuation and future consideration of percutaneous coronary intervention (PCI) to LAD versus minimally invasive coronary artert bypass grafting (CABG) with LIMA to LAD.

## Discussion

 HIV represents a well-established cause for the development of cardiovascular disease (CVD). The underlying pathophysiological mechanism of both HIV infection and antiretroviral therapy is associated with the progression of atherosclerosis, subsequently establishing coronary artery disease.^4,5^ In individuals with HIV, the presence of chronic inflammation and endothelial dysfunction may contribute to an increased susceptibility to vascular damage, potentially including SCAD.^4,5^

 The diagnosis of SCAD can be challenging, because of its resemblance with other causes of ACS, such as atherosclerotic plaque rupture. Coronary angiography alone is not always enough to ensure the diagnosis, because SCAD can be presented with a variety of angiographic results.^6^ In the majority of the cases intravascular techniques, such as OCT usually confirm the diagnosis with the presence of intramural hematoma and a false lumen.^6^ In the present case, IVUS was used to establish the diagnosis.

 Guideline-directed medical therapy for SCAD is initially conservative, involving a wall self-healing process with dual antiplatelet therapy, and beta blockers leading to complete angiographic resolution of the lesion in most cases.^7^ However, optimal medical therapy is limited to expert opinions, due to the absence of clinical trials supporting evidence-based management.^7^ This approach is preferred as revascularization comes with a number of complications. This includes entering the false lumen with the guiding wire, the extension of intramural hematoma, or long-term complications with stent mal-position and thrombosis.^8^ However, there are high-risk situations where revascularization is appropriate, this includes the dissection of the left main coronary artery, a thrombolysis in myocardial infarction (TIMI) grade 0-1 proximally, hemodynamic compromise, and persistent arrhythmia.^7^ In these cases, imaging-guided PCI strategies involving long stent coverage of the dissection, avoiding pre-dilatation, or multi-stenting approaches beginning from the edges and finishing at the middle part of the dissection are recommended.^7^ The decision between medical therapy revascularization (PCI or CABG) should be individualized based on the extent of coronary involvement and the patient’s clinical presentation.^8^

 Abacavir is a nucleoside analog reverse-transcriptase inhibitor that is commonly used in HIV patients, alongside newer drug classes like integrase strand transfer inhibitors and reverse transcriptase inhibitors, for improved efficacy and reduced side effects. Despite its therapeutic value, Abacavir has been implicated in increasing cardiovascular risk.^9^ There is an ongoing debate in the literature, as some of the studies strengthen the hypothesis that there is a connection between abacavir and the development of ACS, while others show no significant risk.^9^ The flagship study was published by the D:A:D study group in 2008, which showed an increased risk of myocardial infarction in patients treated with abacavir.^10^ The reason for the debate is that this study in addition to several follow-up studies, that also supported the initial hypothesis, included a sample of patients with predisposing risk factors to cardiovascular disease, such as hypertension, dyslipidemia, and smoking.^9^ It is worth noting that active smoking likely contributed to the vascular susceptibility and serves as a confounder in the present case. The underlying mechanisms by which abacavir is connected to acute coronary syndrome are still not completely understood. Some possible explanations are: 1) platelet activation that is related to arterial thrombosis^11^, 2) interaction between human leukocytes and endothelial cells which may contribute to vascular damage and progression of atherosclerosis.^12^

 In our case, a significant change in his HIV treatment was made and abacavir was replaced by tenofovir. It has been previously demonstrated that patients switching to a tenofovir based regimen have an improved lipid profile, with a substantial decrease in total cholesterol and LDL levels, thus a reduced cardiovascular risk.^13^ No direct correlation between antiretroviral therapy and SCAD has yet been proven.

## Conclusion

 This case report shows the importance of considering SCAD as a potential cause of ACS in individuals with HIV. It also highlights the need for a comprehensive and multidisciplinary approach to diagnosis and management, considering the unique challenges posed by antiretroviral therapy and the increased cardiovascular risk associated with HIV infection. Further research is warranted to explore the underlying mechanisms of SCAD in this population and to develop reliable treatment strategies.

## Competing Interests

 The authors declare that they have no competing interests.

## Ethical Approval

 An informed consent was obtained from the patient.

## Supplementary Files


Video 1: Initial coronary angiography showing the intracoronary clearance at the site of the dissection

Video 2: Proximal LAD stenosis in the first angiography

Video 3: Follow-up angiography 5 days later showing signs of healing at the dissected area

Video 4: Full OCT run evaluating the healing of the dissection

Video 5: Improved angiographic result in the follow-up angiography 5 months later
